# Supplementary material for: FliH and FliI help FlhA bring strict order to flagellar protein export in Salmonella
Source: Commun Biol. 2024 Mar 26;7:366. doi: 10.1038/s42003-024-06081-0 (PMC10965912; doi:10.1038/s42003-024-06081-0)
Supplement: Supplementary file 2 — Supplementary Information [file 42003_2024_6081_MOESM2_ESM.pdf]

Supplementary Information

FiH and FliI help FlhA bring strict order to flagellar protein export in *Salmonella*

Miki Kinoshita, Tohru Minamino, Takayuki Uchihashi, and Keiichi Namba

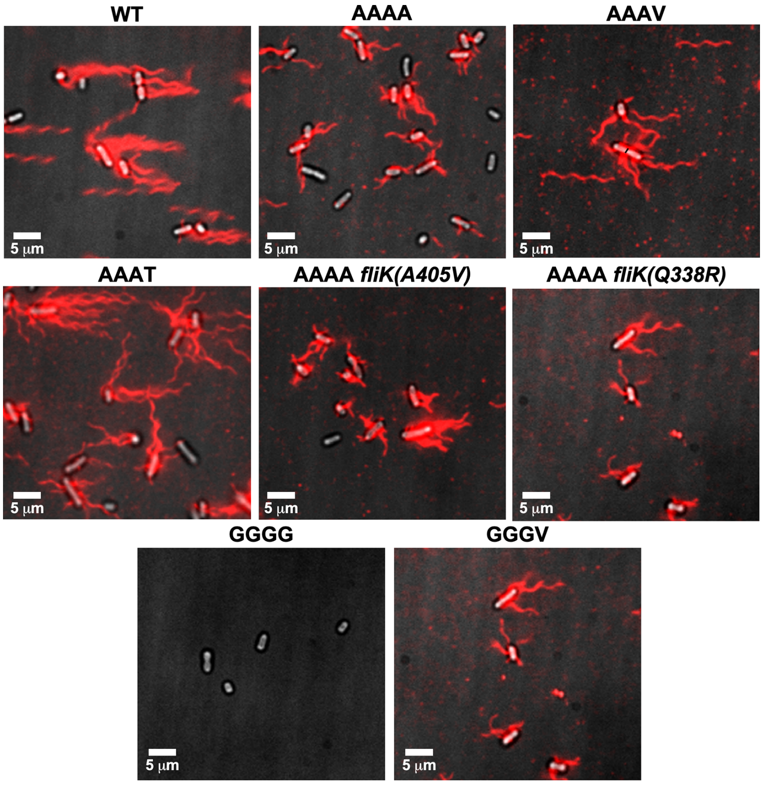

Supplementary Fig. 1. Effect of suppressor mutations on flagellar filament formation in the AAAA and GGGG mutants. Fluorescent images of NH001 carrying pMM130 (indicated as WT), MMA130A4 (indicated as AAAA), MMA130A4-5 (indicated as AAAV), MMA130A4-7 (indicated as AAAT), MMA130A4-3 [AAAA *fliK(A405V)*], MMA130A4-10 [AAAA *fliK(Q338R)*], MMA130G4 (indicated as GGGG), or MMA130G4-3 (indicated as GGGV). Fresh colonies were grown in L-broth containing ampicillin until the cells reached the stationary phase, and then flagellar filaments were labelled with a fluorescent dye, Alexa Fluor 594. The fluorescence images of the filaments labelled with Alexa Fluor 594 (red) were merged with the bright field images of the cell bodies.

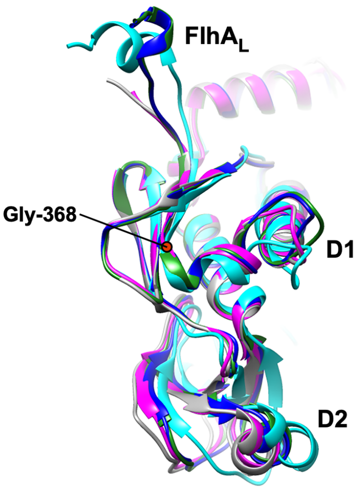

Supplementary Fig.2. Structural comparison of the GYXLI motif in three distinct open, semi-closed, and closed conformations of FlhA$_C$. Ribbon representations of the crystal structures of the open (Mol-A, blue; Mol-B, forest green) (PDB ID: 3A5I), semi-closed (Mol-A, magenta; Mol-B, dark grey) (PDB ID: 6AI0) and closed (cyan) (PDB ID: 3MYD) forms of FlhA$_C$. Domain D1 of each form is superimposed to that of Mol-A of the 3A5I structure.

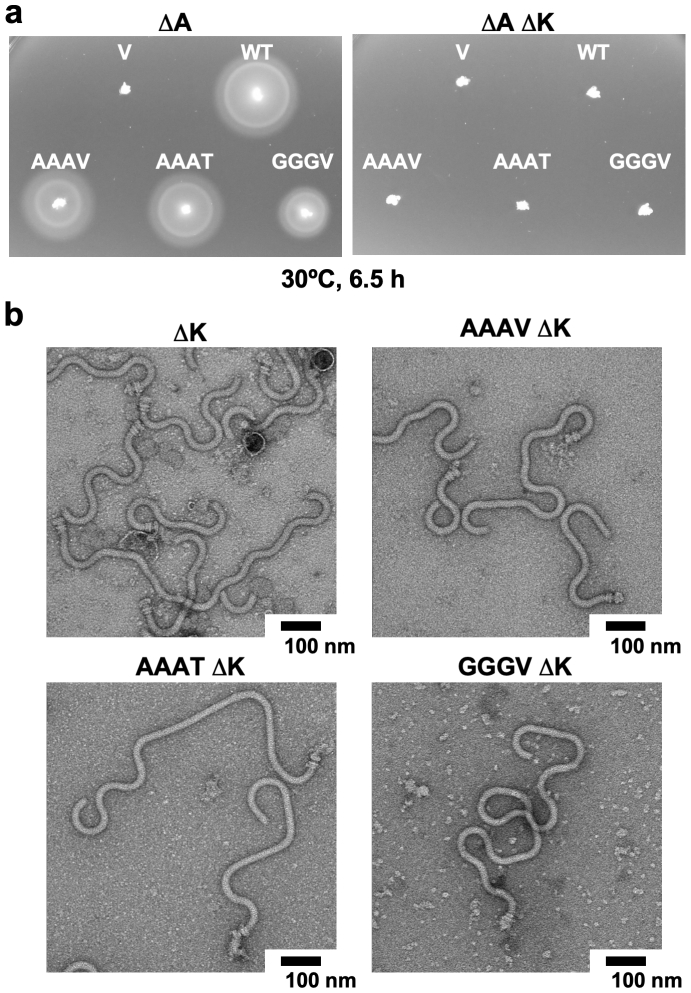

Supplementary Fig. 3. Effect of FliK deletion on the export switching function of the AAAV/T and GGGV mutants. (a) Motility of the *Salmonella* NH001 (Δ*flhA*, indicated as ΔA) (left panel) and NH001iK (Δ*flhA* Δ*fliK*::*tetRA*, indicated as ΔA ΔK) (right panel) strains carrying pTrc99AFF4 (V), pMM130 (WT), pMKM130-A3V (AAAV), pMKM130-A3T (AAAT), or pMKM130-G3V (GGGV) in soft agar. Plates were incubated at 30ºC for 6.5 hours. **(b)** Electron micrographs of polyhook-basal bodies isolated from the above transformants.

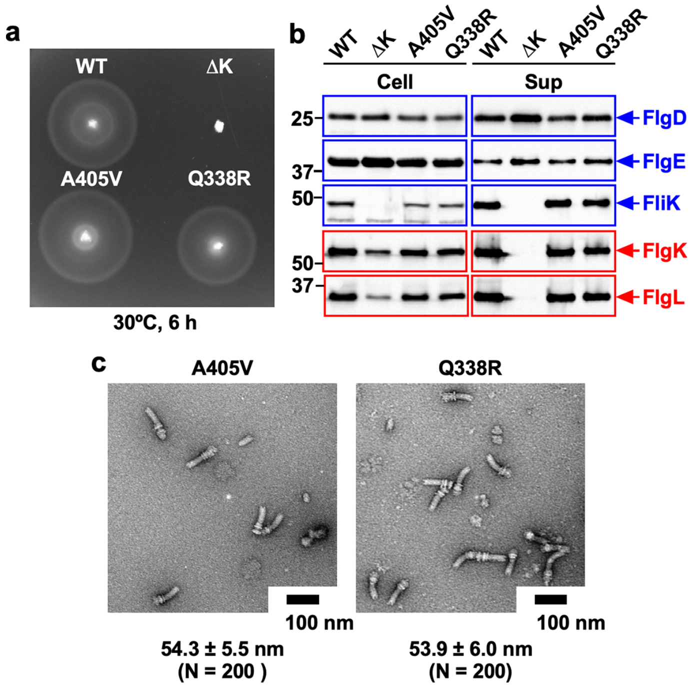

Supplementary Fig. 4. Effect of second site *fliK* mutations on motility. (a) Motility of SJW1103 (wild-type, indicated as WT), TH8426 (Δ*fliK*, indicated as ΔK), MMK130-3 [*fliK(A405V)*, indicated as A405V], or MMK130-10 [*fliK(Q338R)*, indicated as Q338R] in soft agar. Plates were incubated at 30ºC for 6 hours. (b) Immunoblot, using polyclonal anti-FlgD (1st row), anti-FlgE (2nd row), anti-FliK (3rd row), anti-FlgK (4th row) or anti-FlgL (5th row) antibody, of whole cell proteins (Cell) and culture supernatants (Sup) prepared from the above strains. RH-type and F-type substrates are highlighted in blue and red, respectively. Molecular mass markers (kDa) are shown on the left. **(c)** Electron micrographs of hook-basal bodies isolated from the above strains. The average hook length and standard deviations are shown. N indices the number of hook-basal bodies that were measured.

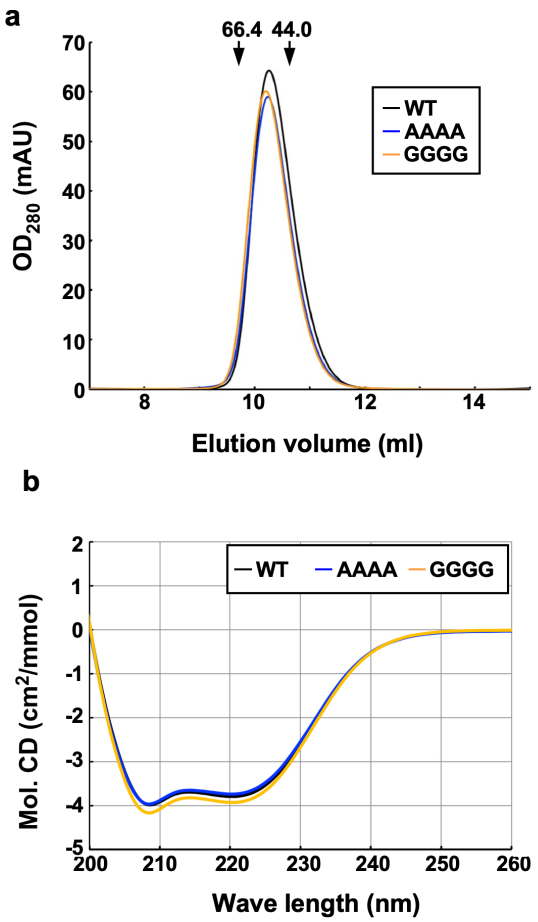

Supplementary Fig. 5. Effect of the AAAA and GGGG mutations on the monomeric FlhA$_C$ conformation. (a) Effect of the AAAA and GGGG mutations on hydrodynamic properties of FlhA$_C$. A 500 µl solution of each purified sample (10 µM) was run on a Superdex 75HR 10/30 column equilibrated with 50 mM Tri-HCl, pH 8.0, 150 mM NaCl. The elution peaks of His-FlhA$_C$ (WT, black), His-FlhA$_{C\text{-}AAAA}$ (AAAA, blue), and His-FlhA$_{C\text{-}GGGG}$ (GGGG, orange) are 10.3 ml, 10.2 ml, and 10.2 ml, respectively. Arrows indicate the elution peaks of bovine serum albumin (66.4 kDa) and ovalbumin (44 kDa), which are 9.7 ml and 10.7 ml, respectively. **(b)** Effect of the AAAA and GGGG mutations on far-UV CD spectra of FlhA$_C$. Measurements were carried out at room temperature in 20 mM Tris-HCl, pH 8.0, in a quartz cell with a path length of 1 mm.

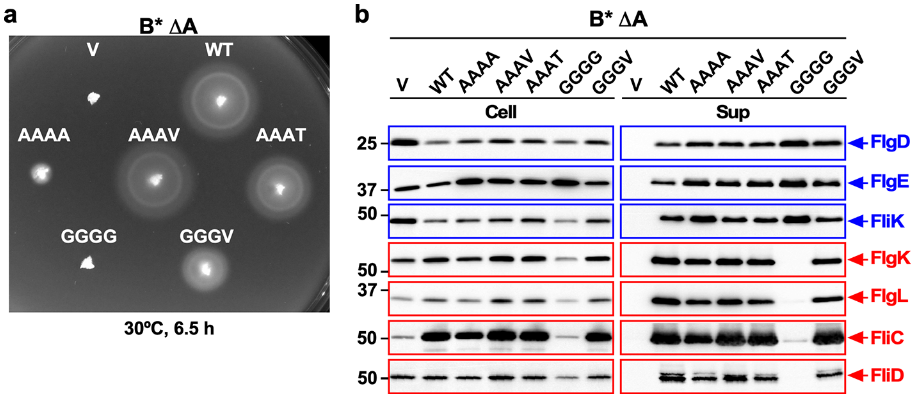

Supplementary Fig. 6. Effect of the *flhB(P28T)* mutation on flagellar protein export by the AAAV/T and GGGV mutants. (a) Motility of the *Salmonella* NH002 [*flhB(P28T)* Δ*flhA*, indicated as B* ΔA] strain carrying pTrc99AFF4 (V), pMM130 (WT), pMKM130-A4 (AAAA), pMKM130-A3V (AAAV), pMKM130-A3T (AAAT), pMKM130-G4 (GGGG), or pMKM130-G3V (GGGV) in soft agar. Plates were incubated at 30ºC for 6.5 hours. **(b)** Immunoblot, using polyclonal anti-FlgD (1st row), anti-FlgE (2nd row), anti-FliK (3rd row), anti-FlgK (4th row), anti-FlgL (5th row), anti-FliC (6th row), or anti-FliD (7th row) antibody, of whole cell proteins (Cell) and culture supernatants (Sup) prepared from the above transformants. RH-type and F-type substrates are highlighted in blue and red, respectively. The positions of molecular mass markers (kDa) are indicated on the left.

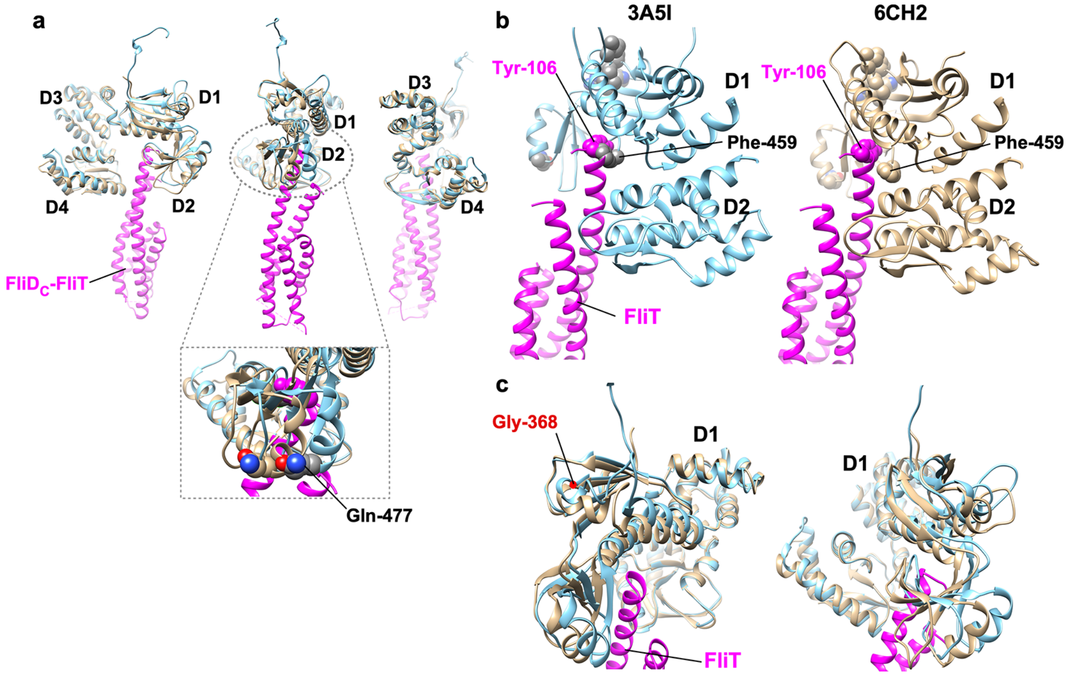

Supplementary Fig. 7. Structural comparison of FlhA$_C$ with and without the FliT chaperone. Ribbon representations of the crystal structures of FlhA$_C$ in an open conformation (sky blue) (PDB ID: 3A5I) and FlhA$_C$ (tan) in complex with FliT fused with the C-terminal region of FliD (magenta) (PDB ID: 6CH2). **(a)** Three different views showing the FliT chaperone binds to the conserved hydrophobic dimple between domains D1 and D2 of FlhA$_C$, resulting in a rotation of domain D2 relative to domain D1 together with a conformational change of the GYXLI motif coupled with a downward shifts of two α-helices of domain D1. Gln-477 is a good indicator of the rotation of domain D2 relative to domain D1. The middle and right panels are rotated 90 degrees clockwise and counterclockwise from the left panel, respectively. **(b)** FliT binds to a well-conserved hydrophobic dimple of FlhA$_C$ located at the interface between domains D1 and D2, and the highly conserved Tyr-106 residue of FliT and the highly conserved Phe-459 residue of FlhA$_C$ are directly involved in the interaction between FlhA$_C$ and FliT. The rotation of domain D2 relative to domain D1 allows Tyr-106 of FliT to fit nicely into the hydrophobic dimple of FlhA$_C$. **(c)** Gly-368 of FlhA$_C$ is located within the conserved GYXLI motif of FlhA$_C$. The YXLI sequence forms a short α-helix. The binding of FliT to FlhA$_C$ induces a conformational change of the conserved GYXLI motif of FlhA$_C$. The right panel is rotated 60 degrees clockwise from the left panel.

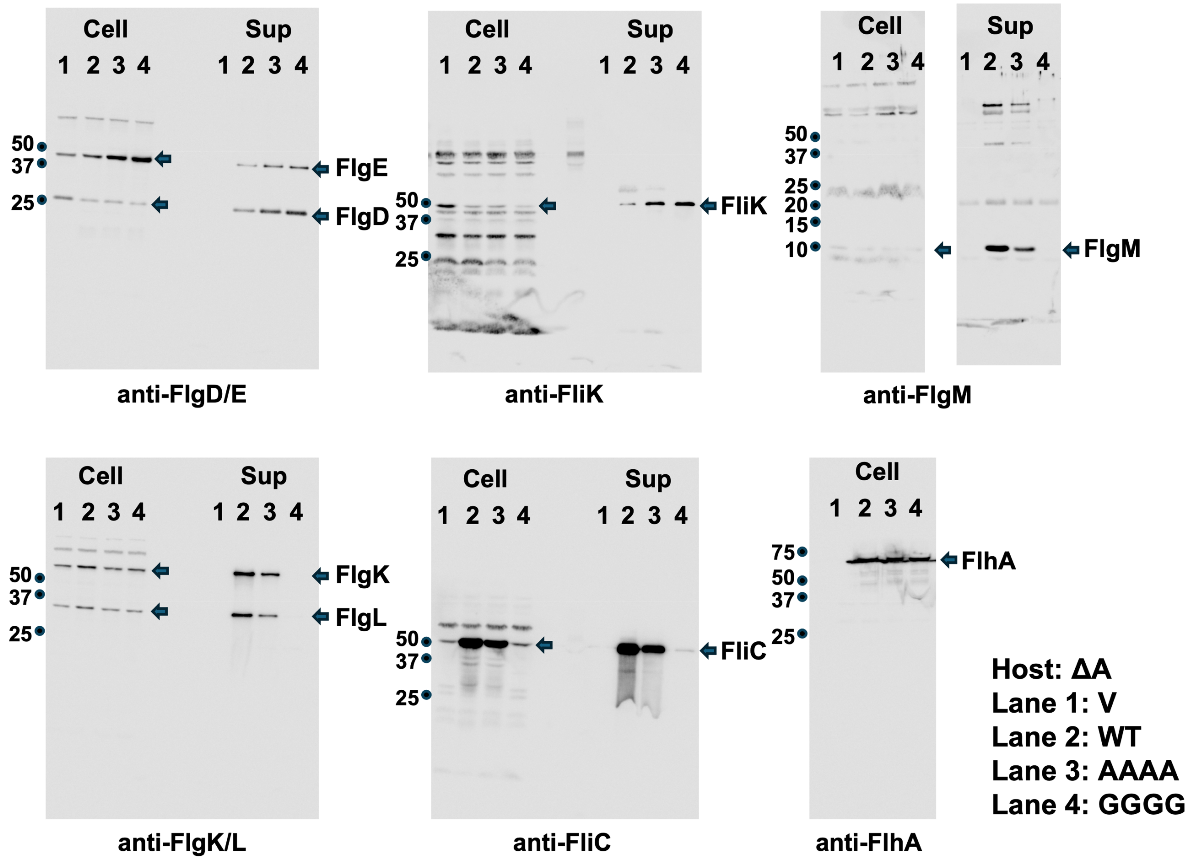

Supplementary Fig. 8. Original immunoblots shown in Fig. 3b. The regions of interest were indicated by arrows. The positions of molecular mass markers (kDa) are indicated on the left.

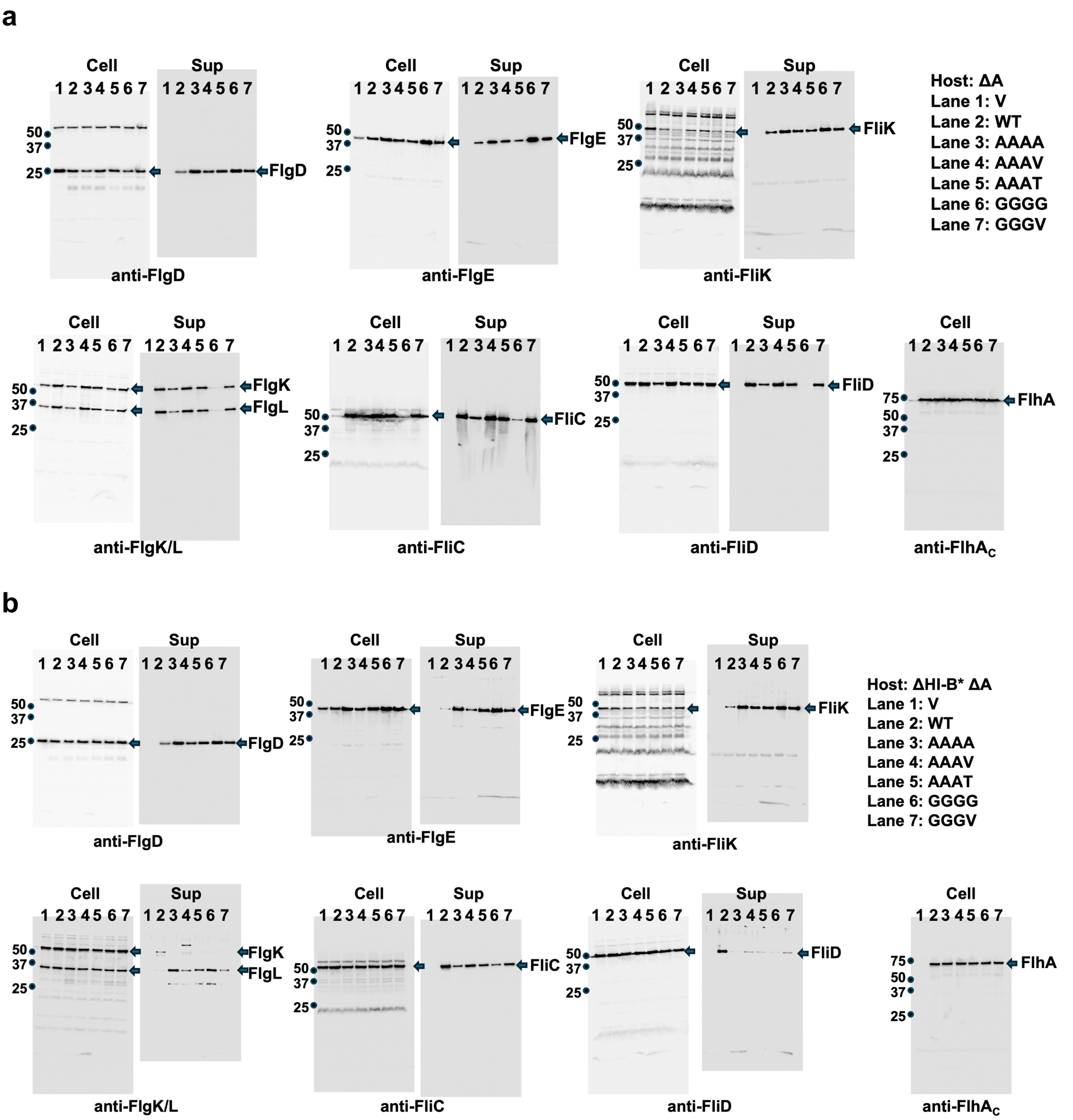

Supplementary Fig. 9. Original immunoblots shown in (a) left and (b) right panels of Fig. 3d. The regions of interest were indicated by arrows. The positions of molecular mass markers (kDa) are indicated on the left.

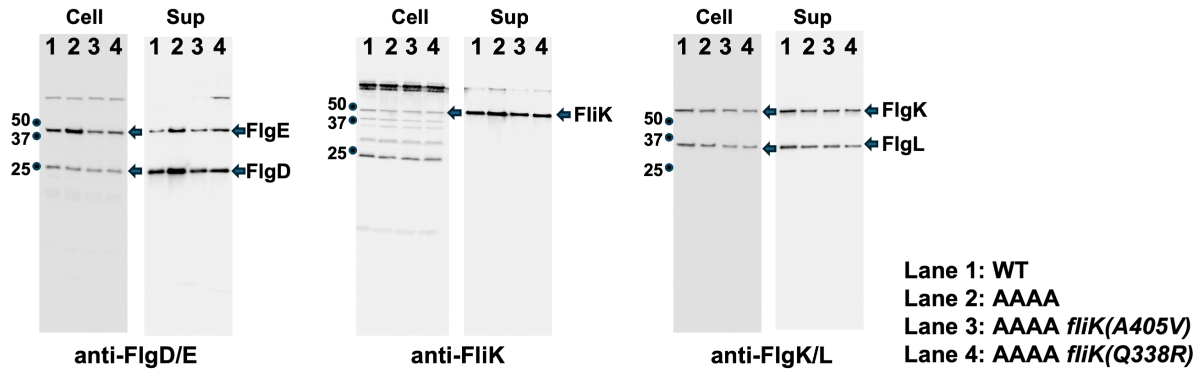

Supplementary Fig. 10. Original immunoblots shown in Fig. 4d. The regions of interest were indicated by arrows. The positions of molecular mass markers (kDa) are indicated on the left.

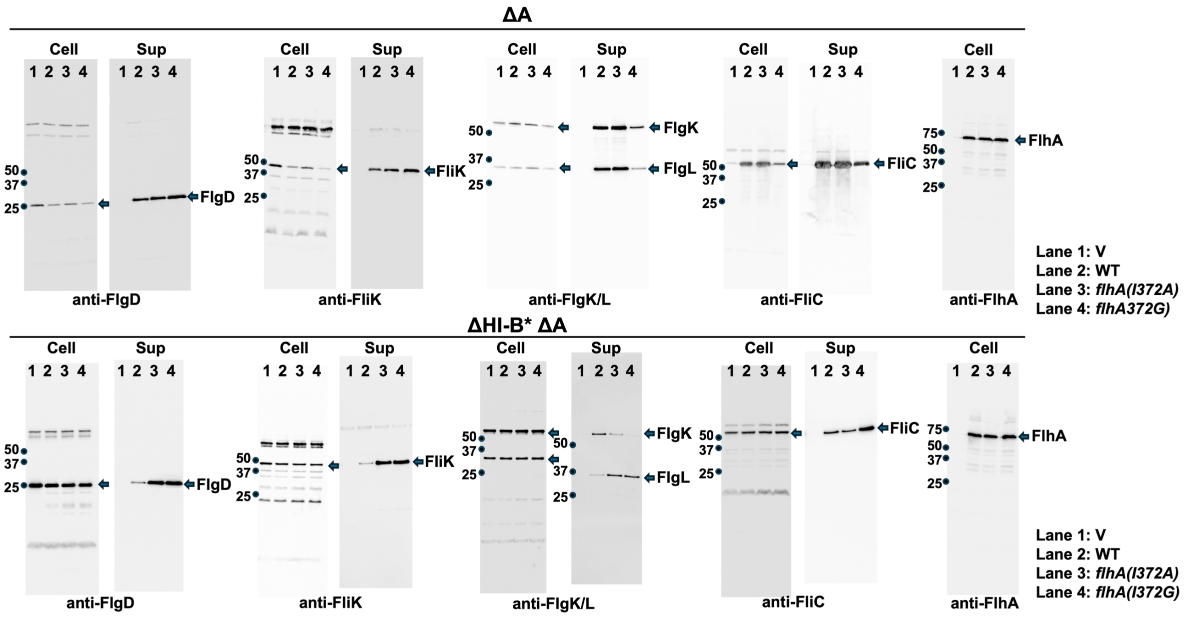

Supplementary Fig. 11. Original immunoblots shown in Fig. 5b. The regions of interest were indicated by arrows. The positions of molecular mass markers (kDa) are indicated on the left.

a

GST-FlgN/FlgK + WT

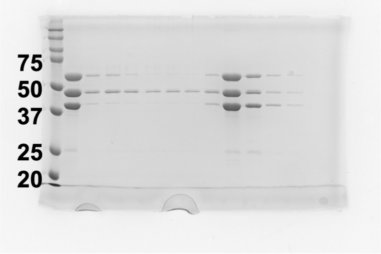

GST-FlgN/FlgK + AAAA

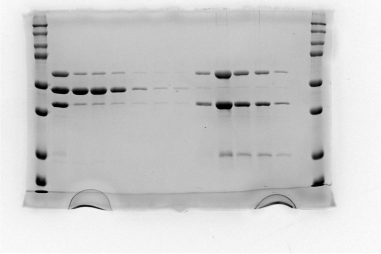

GST-FlgN/FlgK + AAAV

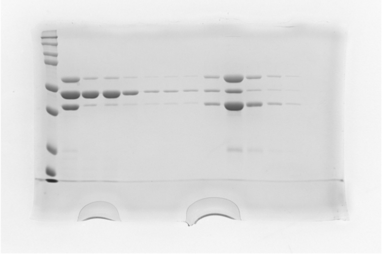

GST-FlgN/FlgK + GGGV

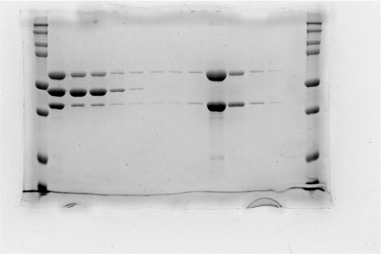

GST-FlgN/FlgK + GGGV

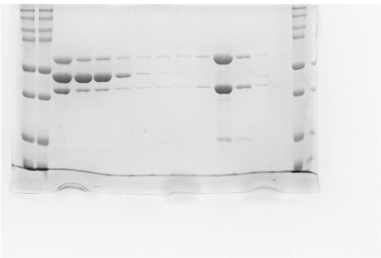

b

GST-FliJ + WT

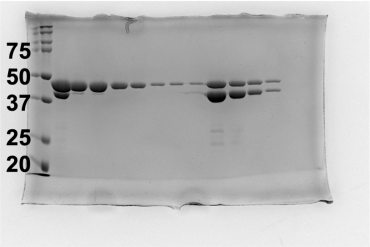

GST-FliJ + AAAA

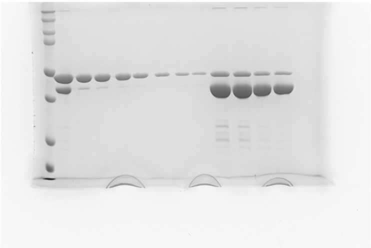

GST-FliJ + AAAV

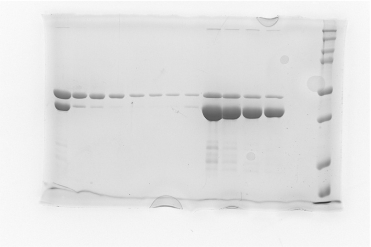

GST-FliJ + GGGG

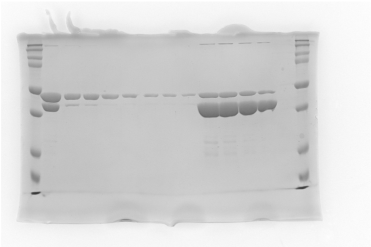

GST-FliJ + GGGV

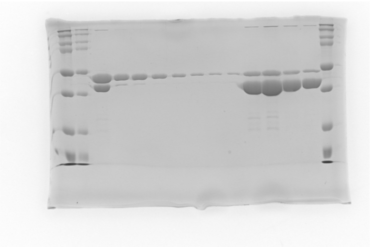

Supplementary Fig. 12. Original CBB-stained gels shown in (a) Fig. 7a and (b) Fig. 7b. Molecular mass markers (kDa) are shown.

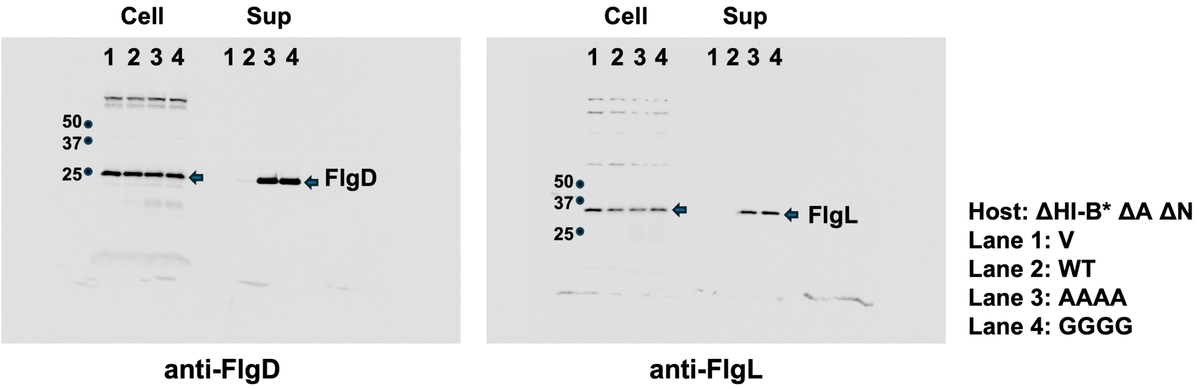

Supplementary Fig. 13. Original immunoblots shown in Fig. 7. The regions of interest were indicated by arrows. Molecular mass markers (kDa) are shown on the left.
