# Supplementary material for: FliH and FliI help FlhA bring strict order to flagellar protein export in Salmonella
Source: Commun Biol. 2024 Mar 26;7:366. doi: 10.1038/s42003-024-06081-0 (PMC10965912; doi:10.1038/s42003-024-06081-0)
Supplement: Supplementary file 3 — Description of Additional Supplementary Materials [file 42003_2024_6081_MOESM3_ESM.docx]

**Description of Additional Supplementary Files**

**File name:** Supplementary Data 1

**Description:** The original data used to measure the length of the hooks
